# A review of surgical procedures to repair obstetric fistula

**DOI:** 10.1002/ijgo.13035

**Published:** 2020-01-13

**Authors:** Rachel Pope, Meghan Beddow

**Affiliations:** ^1^ Division of Global Women's Health Department of Obstetrics and Gynecology Baylor College of Medicine Houston TX USA

**Keywords:** Fistula repair, Global women's health, Obstetric fistula, Vesicovaginal fistula

## Abstract

Although approximately 2 million women suffer from an obstetric fistula, the surgical literature is sparse. This review examines the evidence published to date. The most relevant surgical evidence is included, highlighting the need for further scientific investigations to contribute to our surgical practice. The most pressing needs relate to anti‐incontinence techniques and complex obstetric fistula repairs.

## Abstract 2

This summary of surgical techniques and procedures in obstetric fistula repair demonstrates the need for more evidence and innovation.

## 1 INTRODUCTION

Although obstetric fistula affects an estimated 2 million women worldwide,^1^ the compendium of published surgical research is astoundingly sparse. The oldest evidence of the condition was found in a mummy dating back to 2050 BC.^2^ Written accounts of obstetric fistula have been described, including a 10th‐century description by Ibn Sina, who related it to perineal injuries.^3^ More detailed descriptions of obstetric fistula were recorded in 1597; however, no further mention was made until the next century, when surgeons throughout Europe and the USA competed to devise methods of surgical repair, albeit with limited and rare success.

In 1663, van Roonhuyse of Amsterdam described his technique of denuding the tissues and using suture to close the defect. In 1752, Fatio of Switzerland reported using sharpened quills to reapproximate tissue. A “tension‐free repair” was first hinted at in 1834, when French surgeon de Lamballe described his steps to reduce the strain on the sutures by making wide lateral incisions on the vagina. The cure for a vesicovaginal fistula was described as the “rarest of occurrences” by Deiffenbach in 1845.^2^

Although ethically reprehensible, J. Marion Sims published a successful closure rate of 73% by denuding the edges of the fistula to the bladder mucosa, suturing the fistula closed with silver wire and clamps, and decompressing the bladder with a catheter. Sims’ pupil, Thomas Emmet, published their work in 1868, making one of the first substantive contributions to the medical literature regarding fistula surgery.^4^

Nearly a century and a half later (2020) we continue to function in a field without robust surgical writing and evidence. Many gaps in our practice are due to the unique challenges associated with conducting the studies and work necessary in the settings and populations affected by obstetric fistula. Kelly and Winter^5^ advocate for more clustered randomized controlled trials on obstetric fistula, specifically its treatment; however, they do acknowledge the difficulties in randomization. In addition, iatrogenic fistula seems to be on the rise in many countries where obstetric fistula is prevalent, leading to a need for more research into the prevention and treatment of these injuries as well. The aim of the present article is to review the most relevant current literature on surgical aspects of obstetric fistula, including a delineation of what is still unknown, highlighting the need for further investigations to contribute to refinement of our surgical practice.

## 2 CLASSIFICATION AND OUTCOMES

Using a uniform language to describe a condition enables sharing of medical knowledge and description of a procedure. Frajzyngier et al.^6^ examined prognostic values of classification systems for surgical closure of genitourinary fistula and found them to be “poor to fair.” Goh's classification system predicts the risk of failure in fistula patients with scarring and circumferential defects. It also demonstrates that fistulae near the external urethral orifice (EUO) are more likely to result in residual incontinence.^7^ Waaldijk's classification is based on the effect on the urethral closing mechanism, followed by size, representing increasingly difficult surgical technique from type I to III.^8^ There are various other classification schemes in existence, with unknown prevalence of use.^9^ Designation of one universal classification would assist the comparison of research and clinical outcomes across a variety of settings.

Published rates of successful surgical closure of obstetric fistula are high (80%–97%).^10^, ^11^, ^12^ In a series of 384 patients, Ouedraogo et al.^10^ reported success rates of 92% for “easy” cases, 68% for “intermediate” cases, and 57% for those deemed “difficult.” Repair outcomes have been associated with the duration of the fistula before surgery, fistula size, bladder size, circumferential fistula or those with urethral involvement, HIV status, and moderate to severe vaginal scarring.^11^, ^12^, ^13^

## 3 BLADDER DECOMPRESSION AND TIMING OF CLOSURE

Although specific criteria are lacking, use of a catheter is recommended prior to surgical intervention: in early recognition (less than 1 month following development) to allow for spontaneous vesicovaginal fistula (VVF) healing, or in an individual at high risk for developing VVF after prolonged obstructed labor. Tayler‐Smith et al.^14^ and Waaldijk^15^ reported healing rates of 11% and 15%, respectively, in small, early VVF with prolonged catheterization (6 weeks).

Traditionally, surgeons have waited 3 months after a vaginal delivery to operate, allowing time for spontaneous closure and for granulation tissue to disappear. However, both Waaldijk^16^ and Raassen et al.^17^ documented success with immediate operation on “fresh fistulae” rather than waiting. The closure rates in their samples were 95.2% (n=1633) and 93% (n=91), respectively, at first attempt. Waaldijk^15^ also discussed immediate surgery after a period of catheterization. The catheter remains until the fistula edge is no longer necrotic, at which time the fistula is repaired. Closure rates of 91.8% (n=156) using this timing for repair are also encouraging.

The management of iatrogenic fistula is debatable. Lo et al.^18^ report that iatrogenic fistulae are less likely to close spontaneously with bladder decompression, owing to location and size, and advise early transabdominal closure of this type. A recent meta‐analysis on iatrogenic VVF demonstrated that 8% of patients treated with bladder decompression experienced spontaneous closure.^19^ The same meta‐analysis noted a 93% success rate for vaginal closure, while avoiding the morbidity of an abdominal approach. For juxtacervical fistula, Chigbu et al.^20^ found that there was no difference in outcomes between the abdominal route versus the vaginal route. More information is needed to discern which fistula types and factors would lend to healing with catheterization versus surgery. Operating sooner rather than later has the great potential for reducing stigma and social isolation for women with obstetric fistula.

## 4 SURGICAL TECHNIQUE

Surgical technique is described in multiple textbooks; however, scientific evidence behind technique is lacking. The vaginal approach to repair an obstetric fistula is generally described and preferred by most surgeons.^21^, ^22^ The suture material selected and number of layers used for closure have not been studied in a systematic way; however, retrospective data demonstrate no superiority using two layers when controlled for bladder size.^23^

For circumferential fistula, experienced surgeons stress the importance of circumferential anastomosis.^24^ Urethral fistula and residual fistula were commonly repaired using the Martius graft. However, two retrospective reviews demonstrated no difference in fistula closure using the graft, but an increase in urethral incontinence.^25^, ^26^ Therefore, this procedure should no longer be commonplace in obstetric fistula surgery.

More recently, fasciocutaneous and muscle flaps have been used for vaginal reconstruction and to provide a better chance of continence.^27^, ^28^ As described by Pope et al.,^27^ the blood supply and innervation are maintained in the fasciocutaneous flap (Singapore flap); this technique is reproducible in low‐resource settings, with a relatively short operative time and no need for postoperative molds since it is a true flap, not a graft. However, as a more extensive surgery, patient selection is necessary.

Browning et al.^28^ described success with the Singapore skin flap used prophylactically with Goh type 4ciii fistulae: 46% of women were dry compared with an expected 19%. In a series of women with leakage following previous fistula closure—some of whom had had multiple prior surgeries and significantly distorted anatomy—the Singapore flap provided 71% dryness compared with an expected 26%.^28^

The gracilis muscle flap may be used to augment fistula repair and is now being studied for efficacy in the setting of obstetric fistula. In a study of patients with complex fistulae (i.e. residual and recurrent fistulae, short urethras, small bladders, significant fibrosis, and thin bladder muscularis, all of whom were likely to fail standard fistula repair), 95% of patients were dry immediately following repair using the gracilis muscle flap.^29^ Preliminary follow‐up results show increased continence in patients who received both a Singapore and gracilis flap at time of repair.

The first attempt is still widely considered to be the best chance for success, likely due to tissue and healing factors, which supports the idea of centralization of care in centers of excellence, especially for complex cases.^11^ Women who experience failure of the first repair likely have more complex fistulae, making the first repair less successful and each successive repair more difficult. If a patient requires a repeat procedure it is often performed in the same fashion as the first attempt. However, limited evidence indicates that incorporating a gracilis muscle flap may lead to improved outcomes.^29^ Other innovations for complex fistulae are needed.

## 5 POSTOPERATIVE CATHETERIZATION

Traditionally, a Foley catheter is maintained in place after fistula repair to allow proper tissue healing without tension from bladder distention. The necessary length of catheterization time is debated, and three studies have been published on this. Barone et al.^30^ conducted a randomized controlled trial of 7 versus 14 days for postoperative catheter duration. While no inferiority was determined, this study excluded “not simple cases,” yet included those with previous repairs, which are known to have worse outcomes. Nardos et al.^31^ described 212 patients who had a Foley catheter in place for 10, 12, and 14 days in a study from Ethiopia. The more complicated the fistula, the longer the Foley catheter remained. As breakdown rates for those with catheters in place for 10 days was 1.5%, the authors concluded that this may be sufficient time for simple fistulae. The group catheterized for 12 days had no breakdowns. The authors went on to prospectively randomize patients to 10 and 14 days of catheter duration, excluding circumferential fistula and residual fistula after previous failed repair.^32^ There was no significant difference in repair success, demonstrating the lack of inferiority of 10 days catheterization for fistula repairs that are not repeat surgeries or circumferential.

## 6 URETERIC STENTS

Intraoperative ureteral stent placement is a common procedure among fistula surgeons; however, there is no consensus on the length of time that the stent should remain in situ following the procedure. There must be a balance between the prevention of obstruction/occlusion of the ureters and the risk of pyelonephritis caused by this foreign body. As the morbidity of pyelonephritis may lead to immediate poor outcomes (i.e. sepsis, acute kidney injury) and in the long term to chronic renal insufficiency and hypertension, this is an area of study that is worth exploring further.

## 7 RECTOVAGINAL FISTULA

Rectovaginal fistula (RVF) is also considered obstetric fistula; it is often present concomitantly with VVF and likely represents worse or longer obstructed labor. A true RVF must be differentiated from a chronic fourth‐degree perineal laceration, for which many women will present with similar symptoms. There is little published information on the surgical techniques and outcomes of RVF alone or in the presence of VVF, although there is more literature from high‐resource countries on overall management. In a retrospective study of patients in Ethiopia, Browning and Whiteside^33^ described successful closure with a flap‐splitting technique, avoiding the use of diverting colostomies or grafts.

## 8 ACHIEVING “CURED” STATUS

No standardized definition of “cured” has been established. While this is often considered the same as “dry,” we know that even when a fistula is closed completely, a woman may experience urinary incontinence from intrinsic sphincter deficiency, stress incontinence, or leaking from a variety of other causes.

Browning^34^ found that urethral involvement resulted in an odds ratio (OR) of 8.4 (95% CI, 3.9–17.9) for ongoing incontinence. Other factors leading to ongoing incontinence include a small bladder following repair (<5 cm depth from external urethral orifice; OR 4.1; 95% CI, 1.2–13.8), vaginal scarring (OR 2.4; 95% CI, 1.5–4.0), and larger fistula size—with each centimeter increase in diameter of fistula size, there was an increased OR of 1.3 (95% CI, 1.16–1.56) for ongoing incontinence. There was no association between ongoing incontinence and the length of time elapsed since the delivery that caused the fistula.

While many centers do not examine patients postoperatively at all, a 1‐hour pad weight test may be useful in determining true “cure,” as the source of the leakage often does not matter to the patient. However, a dye test and pad weight can differentiate between a leaking fistula and urethral leakage and therefore inform patient management.

Kopp et al.^35^ examined 346 women after fistula repair to determine a significant pad weight for incontinence. They determined that a 1‐hour pad weight after catheter removal of less 1.5 g had a positive predictive value of 94% (95% CI, 90.0–96.9) in predicting ongoing continence.

## 9 ANTI‐INCONTINENCE PROCEDURES

Anti‐incontinence procedures after fistula repair have not been shown to greatly improve continence status. The most common techniques include autologous fascia slings (i.e. fascia lata and rectus fascia), vaginal pubococcygeal slings, and synthetic slings. The rate of erosion with synthetic slings is unacceptably high, and while autologous fascia slings may help some women, they are also at risk of erosion.^36^, ^37^ Browning^38^ described a prophylactic fibromuscular sling (pubococcygeal sling) used to treat patients with Goh 3 and 4 fistulae (involving the urethra) and larger than 1 cm; there was a decrease in urethral incontinence from 55% (n=44) in patients without the sling to 39% (n=272) in patients with it.

Pope et al.^39^ published a randomized controlled trial comparing prophylactic rectus fascia slings with pubococcygeal slings for individuals at highest risk for residual stress incontinence. Results for rectus fascia slings were promising, although long‐term follow‐up was a challenge, resulting in no difference between the slings. An autologous sling that maintains its vascular supply could potentially improve upon these outcomes.

Both periurethral and autologous fat bulking have been explored for women with urethral incontinence after obstetric fistula repair; however, the results are limited to 2 weeks after the procedure**.** 40, 41 Urethral plugs have been used widely as an alternative therapy to additional surgery.^42^ Plugs have been found to help 75.7% of women leaking urine from the urethra after successful fistula repair^42^; however, the manufacturer of the most commonly used urethral plug has ceased production, resulting in many women without further access to this modality.

## 10 VAGINAL RECONSTRUCTION

Vaginal reconstruction is also likely to be necessary to achieve “cure” from the symptoms of obstetric fistula. Wall et al.^43^ estimated that approximately 30% of fistula patients require vaginoplasty at the same time as surgery owing to lack of adequate healthy vaginal tissue or distorted anatomy from the amount of scarring that has taken place. A recent study found that around one‐third of patients felt that intercourse returned to its pre‐fistula state after surgical repair.^44^ In this cohort, 12% (n=14) experienced de novo sexual dysfunction, half of whom attributed this to pain and half to ongoing incontinence during intercourse. A fistula of more than 3 cm in diameter and a decreased vaginal caliber were associated with sexual dysfunction postoperatively. Only one paper thus far has documented reconstructive techniques.^27^

## 11 URINARY DIVERSION FOR IRREPARABLE FISTULA

The ethics surrounding urinary diversion have been debated owing to a series of unknown variables surrounding follow‐up and long‐term outcomes—significant morbidity and mortality can result from these procedures. The outcome of an international symposium on the topic suggested that only expert fistula surgeons are qualified to deem a woman irreparable by traditional standards, and the procedure should only be offered if appropriate long‐term medical follow‐up is possible.^45^

## 12 CONCLUSION

The repair of obstetric fistula, though studied for hundreds of years, is a subset of surgical expertise that lacks evidence‐based guidance. Obstetric fistulae are a heterogeneous group, with a variety of surgical approaches and outcomes yet to be described. Many experiences of expert surgeons are not submitted for publication, therefore limiting accessibility. An effort must be made to share best practices among the surgeons who care for these women to advance our field and offer the procedures with the best possible outcomes. Continued commitment to sharing ideas, innovations, and experiences will help to move our field forward and provide the highest quality care to some of the most vulnerable women in the world—those living with obstetric fistula.

## AUTHOR CONTRIBUTIONS

RP conceived the article's concept. RP and MB drafted and edited the manuscript.

## CONFLICTS OF INTEREST

The authors have no conflicts of interest.
